# High-risk pregnancy identification and management through antenatal, intrapartum, and postnatal care: an implementation research study protocol

**DOI:** 10.3389/fgwh.2026.1656726

**Published:** 2026-02-06

**Authors:** Mukti Khetan, Rakhi Dhankhar, Ramesh Kumar Huda, Ramesh Kumar Sangwan, Bontha V. Babu

**Affiliations:** 1 ICMR-National Institute for Implementation Research on Non-Communicable Diseases, Jodhpur, India; 2 Indian Council of Medical Research, New Delhi, India

**Keywords:** postnatal care, antenatal care, continuum of care, high-risk pregnancies, implementation research, intrapartum

## Abstract

**Introduction:**

As per WHO, in 2023 more than 700 women died every day from preventable causes related to pregnancy and childbirth. Early identification and management of high-risk pregnancies are essential for improved maternal and child health outcomes and reduced mortality rates.

**Objectives:**

The study objectives are: (a) To identify the barriers and facilitators in implementing existing national programs and the continuum of care for high-risk pregnant women. (b) To co-develop, optimize and implement a context-specific, scalable, and sustainable model of implementation strategies that will help achieve population-based coverage of high-risk pregnancy identification and management. (c) To evaluate the effect of the optimised and contextualised implementation model on the key performance indicators (d) To document the processes of evolution implementation, adoption, adaptation and fidelity of the implementation model.

**Methods:**

The study will utilize the Consolidated Framework for Implementation Research and will be implemented in Nagaur district of Rajasthan, India. The study involves formative research and situational analysis, as well as developing a package of implementation strategies through an iterative process of concurrent implementation, evaluation, and model refinement based on programme learnings. The intervention package will include refresher training of ASHA/ANM workers, strengthened community awareness, and monthly Pradhan Mantri Surakshit Matritva Abhiyan specialist camps.

**Expected outcome:**

The study is expected to improve the identification and management of high-risk pregnancies and enhance the quality of institutional deliveries by strengthening the continuum of care across all stages of pregnancy. Thus, the study is expected to improve maternal and child health outcomes by developing sustainable strategies for scaling up evidence-based, context-specific interventions.

**Implication:**

The study will generate a scalable implementation model that can be adopted more widely to streamline high-risk pregnancy care and contribute to long-term improvements in maternal and child health outcomes.

## 1 Introduction

High-risk pregnancies (HRPs) and associated maternal mortality constitute a significant healthcare challenge in India. It is reported that 1.3 million women have died due to pregnancy-related complications in the last two decades (1). The situation is more alarming in some high-focus states such as Rajasthan. The Maternal Mortality Ratio in Rajasthan is 113 deaths per 100,000 live births, whereas in India, is 97. Similarly, the Infant Mortality Rate in Rajasthan is 32 per 1,000 live births, while it is 28 for India. The major causes of maternal death are pregnancy-related infection, severe bleeding, hypertension, and gestational diabetes, which all come under HRP factors.

According to the fifth National Family Health Survey-5 (NFHS-5) (2), 49.4% of Indian women experienced HRP, of which 33% faced a singular high-risk situation, and 16.4% encountered multiple high-risk pregnancies (3). Despite this high burden, service utilisation remains suboptimal; only 39% of women accessed essential services such as antenatal care, institutional delivery, postnatal care, and complete immunisation for their children (NFHS-5). This highlights the requirement for a continuum of care to ensure optimal health outcomes for mothers and newborns.

The government of India has introduced various programs like the the Pradhan Mantri Surakshit Matritva Abhiyan (PMSMA) launched in 2016 to identify and manage HRPs by providing comprehensive antenatal care (ANC) services to pregnant women throughout India (4).

Another initiative, the Pradhan Mantri Matru Vandana Yojana (PMMVY) supports pregnant women and lactating mothers financially. Under this scheme, the amount (Rs. 5,000) is received in instalments to improve the health and nutrition of the mother and child, and also to compensate for wage loss during pregnancy. It was first launched in 2010 and renamed in 2017 (5). The Labour Room and Quality Improvement Initiative (LaQshya), introduced in 2017, focuses on improving facilities, training healthcare providers, and implementing best practices to ensure safe childbirth for HRPs (6). In 2019, the Surakshit Matritva Aashwasan (SUMAN) was launched to ensure access to emergency obstetric care and other essential interventions for HRPs, safeguarding maternal and fetal health (7).

However, gaps in implementation persist. For instance, literature indicates that approximately 40% of surveyed women were identified as having HRPs in Etawah, Uttar Pradesh, and Belagavi, Karnataka (8, 9), whereas only 14% of cases were reported through the Pradhan Mantri Surakshit Matritva Abhiyan. Similarly, a district-wide digital Accredited Social Health activists (ASHAs)/Auxiliary Nurse midwives (ANM) tracking program in Chamba increased HRP identification from 3.5% to 27.9% within one year (10).

Given these challenges, the current study project aims to design effective and innovative strategies to implement these existing national programs aims to bridge gaps in the identification and continuum of care of HRPs. By adopting a comprehensive approach, the project aims to identify factors contributing to high-risk pregnancies and address the specific needs of these pregnancies through screening, counselling, management, and appropriate referrals, ultimately improving maternal and neonatal health outcomes.

## 2 Methodology

### 2.1 Study design

The proposed implementation research (IR) will follow a mixed-methods approach (qualitative and quantitative). However, unlike traditional quasi-experimental design, which examines and compares the baseline and endpoint of a single-time intervention, this study will be conducted following an implementation research framework that examines the changes in the implementation model and process indicators over time through careful, systematic observation and through comparing to prior performance. The Reach, Effectiveness, Adoption, Implementation, and Maintenance (RE-AIM) framework shall be adopted for the impact and process evaluation (11).

**Figure 1:**
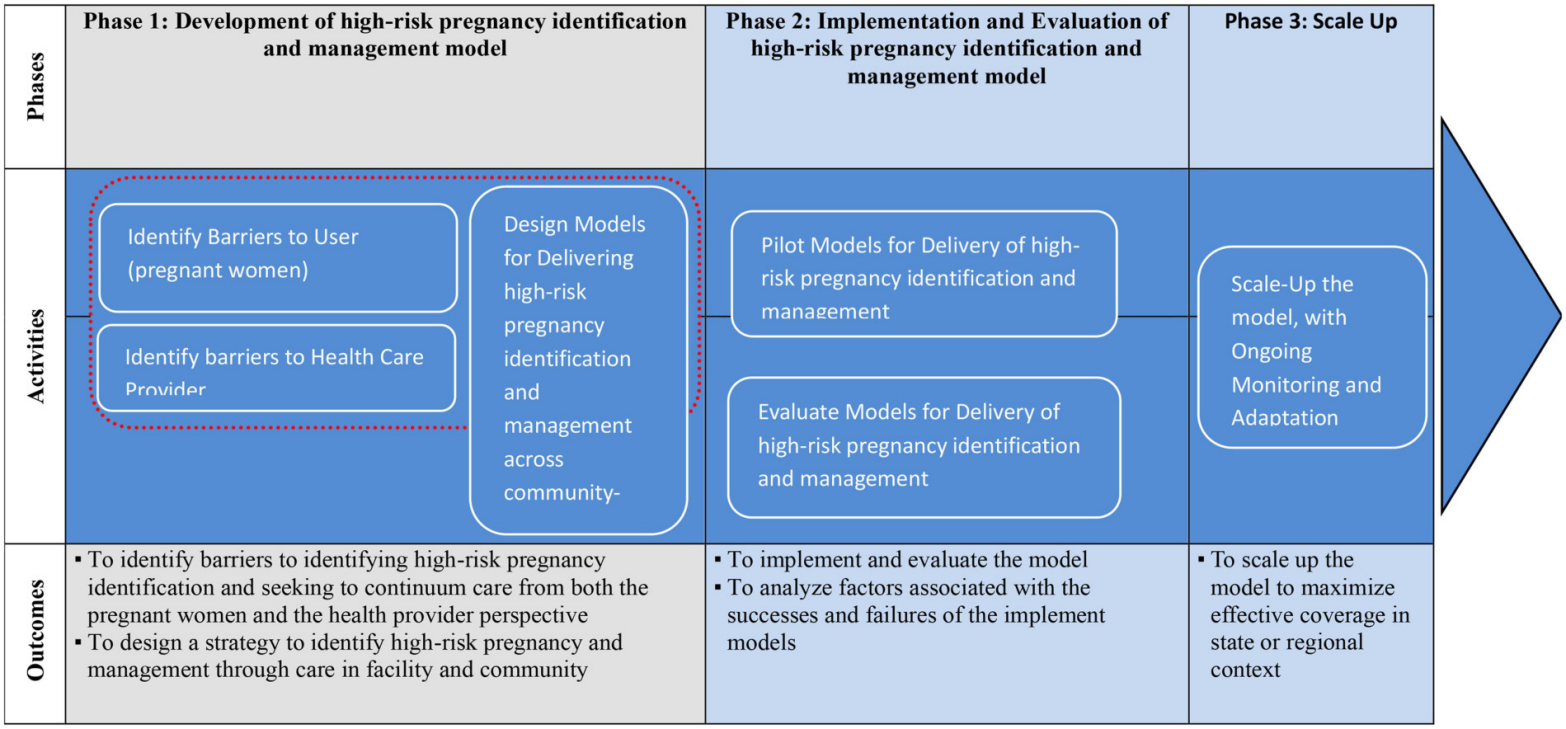
Logic model for high-risk pregnancy identification and management.

Study Phases: The study will be implemented in four phases (Figure 1):

- Preparation, formative and baseline assessment phase
- Co-development, piloting and optimisation phase
- Full-scale implementation phase
- Evaluation phase

### 2.2 Study site

All the sub-centers under three PHCs of Nagaur district of Rajasthan. Estimated expected pregnancies in the three PHCs is around 2,500 per year [based on registration in Pregnancy, Child Tracking & Health Services Management System (PCTS) application between April 2022–March 23]. These sub-centers are located in rural, semi-arid areas where villages are spread out and transport is limited. Each sub-center serves about 5,000 people and is managed by one ANM with support from ASHAs, who identify pregnant women, make home visits, and bring them for check-ups. Each PHC covers around 30,000–35,000 people as per government guidelines and has a team that includes a medical officer, 3–4 Staff Nurses, 1–2 ANMs, a lab technician, a pharmacist, a data entry operator, and support staff. These PHCs provide routine ANC services, basic tests, and referrals, but they often face challenges such as limited specialist services, staff shortage and fewer diagnostic facilities.

### 2.3 Study participants

To identify HRP, the study participants must be pregnant women reporting to the ANC clinic. High-risk pregnant women will be identified using the PCTS application. PCTS will flag HRP cases based on entered clinical and obstetric parameters, ensuring uniform identification across sub-centres. All relevant ASHA, ANM, and healthcare workers are also study participants in the study area. A single standard questionnaire (separate for ASHA, ANM and pregnant women) will be used across all sites and to ensure consistency, all project staff will receive focused training for conducting the interview/FGD.

### 2.4 Sample size: the details of the sample size is given below

#### 2.4.1 Sample size for qualitative data

The sample size for this study will be determined by reaching saturation through in-depth interviews and focus group discussions. A minimum number of eight ANM, eight ASHA and eight pregnant women will be sampled. Sampling for qualitative research will be purposive. Semi-structured questionnaires will be designed separately for pregnant women, ASHA and ANM. Informed consent will be obtained from all the participants and the interviews will be audio-taped for analysis.

Tools/Activities: CFIR framework, FGD guides, and interview guides.

#### 2.4.2 Sample size for quantitative data

Estimating the HRP with prevalence and precision (for baseline assessment): The sample size is calculated considering an anticipated prevalence of high-risk pregnancy (PMSMA) to be 14%. With a relative precision of 10%, a significance level (α) of 0.05, and a design effect of 1.2, 1,925 participants will be needed. To account for 10% possible non-response/refusal or migration, approximately 2,120 women will be surveyed in each round from the catchment areas of the three selected PHCs.

Estimating the change in the HRP proportion between baseline and endline: It is anticipated that a change in HRPs will be detected between the baseline (14%) and endline periods (30%). The sample size needed to detect this change in the HRP detection with via significance level (*α*) of 0.05, a power of 80% is 125 per group (per round of data collection). To compensate for an expected 10%–15% loss to follow-up (primarily due to out-migration of families, refusal to participate in endline survey, miscarriage/stillbirth, or delivery outside the study area) we will recruit about 140–150 participants from each PHC area (from all sub-centers) should effectively capture changes in the HRP detection status. The total sample size will be 450, encompassing all sub-centers under the three PHCs.

### 2.5 Intervention

The intervention will comprise the provision of population-based coverage for HRP identification and management through antenatal care and care continuum across antenatal, intrapartum and postnatal care. The package components will be from the existing program, especially PMSMA, PMMVY, LaQshya, and SUMAN.

The implementation research will deliver a practical package across the three selected PHCs, consisting of (a) refresher training for all ASHAs and ANMs, (b) strengthened facility- and community-level awareness activities for pregnant women and their families, and (c) regular monthly PMSMA camps supported by visiting obstetrician/gynaecologists. For the training of ASHA and ANM workers a dedicated training module on the identification and care of high-risk pregnancies will be developed with the help of experts. In addition, IEC materials on government schemes, antenatal care, and postnatal care will be developed to support PHC and community-level awareness activities.

The key focus areas include:

**Antenatal care:** Early registration, ANC visits, tests and assessments for HRP, and the management of high-risk pregnancies.
**Intrapartum care:** Essential care during childbirth for HRP.
**Postpartum care:** Hospital and home/community care for high-risk pregnancies.

#### 2.5.1 Outcomes

The outcome will be improved population-based coverage of HRP identification and management. The enhancement of data management systems will focus on the facility and ASHA community levels. The outcome involves improving pregnancy surveillance for ASHAs by providing training and support to track pregnant women from identification through delivery and into the postpartum period. Coverage data (population-level) will include proportion of pregnant women identified by ASHA in first trimester; proportion receiving first ANC in first trimester; proportion with 4+ ANC visits; proportion receiving TT injection and 180 IFA tablets. Institutional data (facility-level process) will cover the proportion receiving all planned check-ups; proportion of high-risk pregnancies identified/monitored/referred; proportion of deliveries using safe birth checklist; proportion of HRPs receiving appropriate care; referral timeliness and postpartum contact completion. Coverage data will be gathered directly from ASHAs, while facility data will be streamlined through collaboration with government stakeholders. The research implementation support team will provide guidance and mentorship to government partners. An independent outcome measurement team will also autonomously collect data from pregnant women.

#### 2.5.2 Strategy for high-risk pregnancy identification and management model implementation

**Figure 2:**
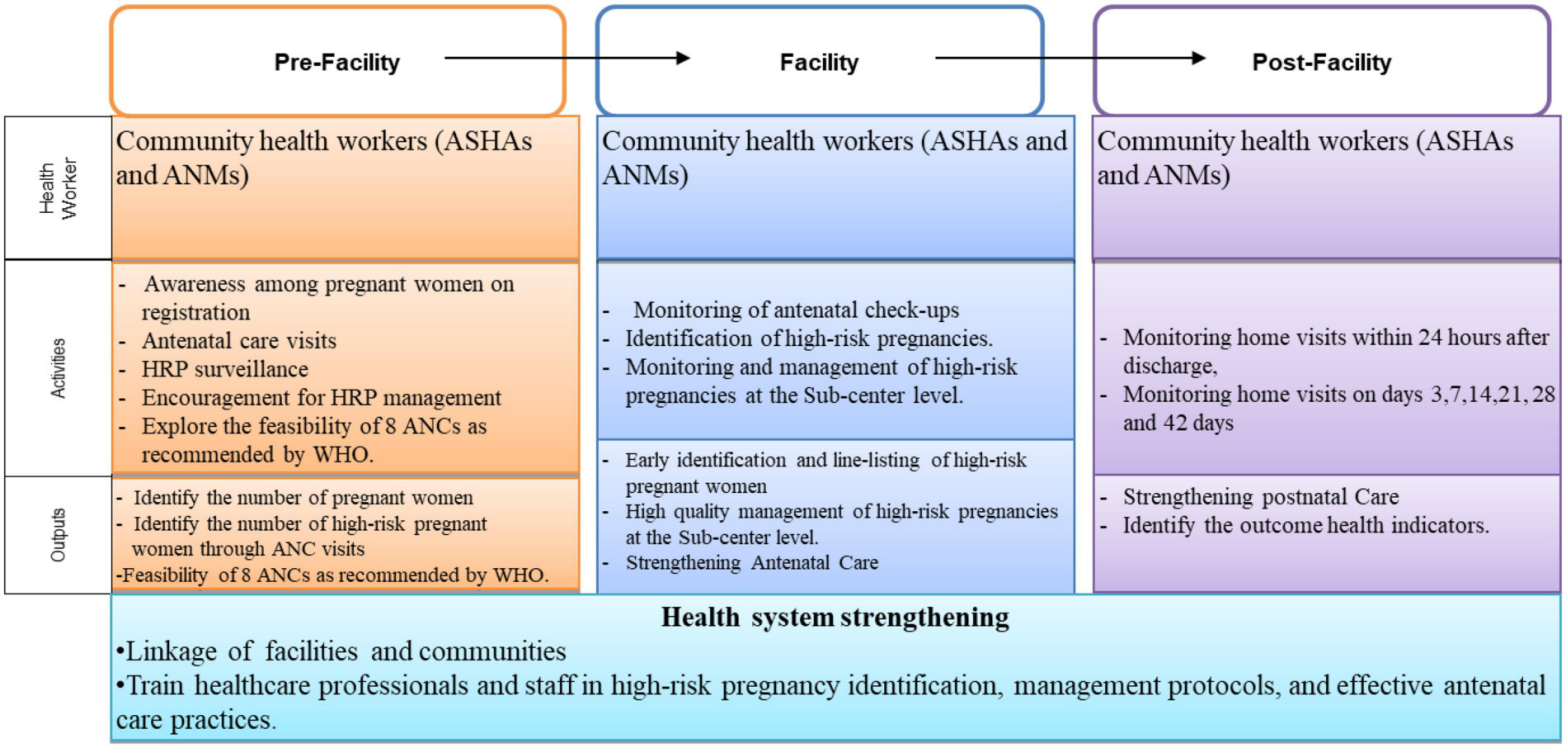
Core framework for implementation with RE-AIM approach.

The Core Framework for achieving high population-based coverage of HRP identification and management strategy will be implemented across three levels (Figure 2):

***Reach (Individual Level):*** Measure the participation percentage of pregnant women in registration and attendance at ANC check-ups for identification and management of high-risk pregnancies.
***Effectiveness (Individual Level):*** Assess the intervention's effectiveness by evaluating improved awareness levels among pregnant women, quantifying increased registration rates for HRPs, and tracking the number of ANCs. Qualitative exit interviews will be conducted after the end line survey to assess satisfaction among pregnant women. A qualitative component will also be included, with in-depth interviews conducted during the formative phase to understand perceptions of care and information gaps. Together, these approaches will enable evaluation of both knowledge improvement and the perceived quality of care.
***Adoption (Staff, Setting, System, or Policy/Other Levels):*** Evaluate how well healthcare facilities integrate the monitoring of antenatal check-ups for high-risk identification and management into existing systems and policies.
***Implementation (Staff, Setting, System, or Policy/Other Levels):*** Evaluate the effectiveness of the comprehensive framework in identifying and managing HRPs across different levels—pre-facility, facility, and post-facility.
***Maintenance (Both Individual and Staff/Setting/System/Policy Levels):*** Confirm the sustainability of the intervention by ensuring that awareness campaigns, antenatal check-ups for high-risk identification and management, and post-facility care remain effective and are integrated into routine healthcare practices.

### 2.6 Study team

The following teams will operate at different levels to implement the IR.

#### 2.6.1 Implementation research team (project team)

The implementation research team will consist of investigators and project team members, organized under three exclusive teams with specific functions -

Implementation Support Team: This team will have the following key functions:(a) Support and facilitate service delivery and community mobilization through the implementation team and (b) improve reporting mechanisms.

Rapid Learning and Feedback Team: This team will perform the following key functions: (a) Systematically review records, observe, and document delivery of all components of interventions (b) Provide routine feedback to implementers on successes and challenges, closely monitoring the progress and compliance of SOPs (Standard Operating Procedures).

Understand the implementers' perceptions of challenges, potential solutions, and other feedback.

Program Evaluation/Outcome Documentation Team: This team will carry out the following key functions: (a) Conduct the baseline assessment (b) Conduct the assessment of facilities, evaluate resource availability, service providers' skills, etc. (c) Conduct an end-line assessment to measure the impact of the implementation research. (d) Conduct concurrent evaluations every quarter.

#### 2.6.2 Implementation team

The implementation team will consist of health system functionaries and managers, who are generally involved in the regular implementation of programmatic and health activities in the district/area (including administrators, teams engaged in program management, care provision/services at facilities and community levels, supervisors, logistics management, data handling and others), and who will continue these activities beyond the period of the IR. State-level teams and officials may also support different implementation components.

### 2.7 The conceptual framework for the proposed implementation research

The IR will use two frameworks for the proposed implementation research.

- The first framework is the Consolidated Framework for Implementation Research (CFIR), which comprises the appropriate domains and constructs of determinants hypothesized to influence implementation outcomes (12).The CFIR has five domains (Innovation/intervention, inner setting, outer setting, process and individuals). We will select appropriate constructs from each of the five subdomains and translate them into inputs for the situational understanding of the existing ecosystem during the formative phase, including (a) assessment of the facilitators and barriers, (b) identification of domains/indicators for data collection during the implementation phase; and (c) to consolidate observations on the implementation process retrospectively at the end.
- The evaluation of implementation strategies and the models developed and implemented shall be done using the RE-AIM framework (11).

### 2.8 Presumptive implementation strategies

We will refer to The Expert Recommendations for Implementing Change (ERIC) discrete implementation strategies list and select and test strategies to adapt, optimize and sustain the interventions (13). Out of the listed ERIC strategies, we will utilise most suitable strategies to deliver the intervention package systematically. However, as needed, new strategies can be opted from the ERIC list during the project's lifetime. Some strategies are given below with standard ERIC nomenclature, and activities proposed under each strategy are delineated (Table 1).

**Table 1:** Summary of presumptive ERIC-based implementation strategies.

ERIC strategy	Planned activities
Build a coalition	The team will involve partners and build relationships with district and block administration, health leadership, non-health departments, private health systems, CBOs, SHGs, and NGOs. Stakeholder mapping and engagement activities will be carried out.
Work with academic institutions	Medical colleges will support strengthening clinical skills, conducting refresher trainings, and improving communication and mobilization capacities. They will also help co-develop the blueprint for implementation strengthening.
Assess readiness and identify barriers & facilitators	Formative research and baseline assessments will be conducted to review existing evidence, assess district preparedness, identify family, community, and system-level bottlenecks, and document key proximal indicators.
Develop a formal implementation blueprint	National guidelines will be contextualized through co-development workshops with district/state leadership, program officers, MOICs, nurses, frontline workers, and community representatives. A draft blueprint with timelines, milestones, and performance measures will be created.
Stage implementation scale-up	Implementation will begin with a 6-month pilot in limited geography, followed by an 18–24-month full-scale rollout across all PHCs. The pilot and full-scale phases will be closely observed, and necessary modifications will be incorporated.
Conduct cyclical small tests of change	During full-scale implementation, small tests of change will be conducted using PDCA/PDSA cycles (3–4 months) to refine strategies before expanding them system-wide.
Increase demand	Strategies will be implemented to generate awareness and increase pregnancy registration and ANC visits by strengthening existing platforms and integrating innovative approaches. The implementation team will support ASHAs and ANMs in mobilizing and engaging pregnant women and their families through regular awareness activities at the PHC and in the community. Project staff will help plan these activities, provide IEC materials, and join selected sessions to ensure clear and consistent communication.
Organize and strengthen quality monitoring systems	Monitoring systems will be organized and harmonized, with timely data collection optimized. Essential and feasible indicators for quality and progress monitoring will be jointly developed.
Identify and prepare champions	A system for recognizing and felicitating champions at program, facility, and community levels will be supported.
Intervene with end-users	ANMs at Subcenters will follow up with pregnant women and families. ASHAs (and AWWs) will identify and mobilize pregnant women.
Strengthen community ownership and accountability	Strategies will be co-developed to promote community ownership of HRP identification and management and to strengthen social accountability mechanisms. As part of this effort, regular community-level awareness camps will be organized with the support of ASHAs, ANMs, and project staff to inform families about HRP signs, available services, and the importance of timely ANC visits.
Capture and share local knowledge	The learning and feedback team will document local knowledge, success stories, and practical solutions and will share them through dissemination and experience-sharing meetings.

### 2.9 Evaluation

The Evaluation will be carried out in two ways:

#### 2.9.1 Evaluation framework

The evaluation of implementation strategies and the models developed and implemented shall be done using the Implementation Research Logic Model (IRLM) and the formative evaluation approach for documenting the qualitative and qualitative components, respectively (14). The IRLM shall enable capturing the relationships between determinants of implementation, implementation strategies, the mechanisms of action, and the implementation and the outcomes. The IRLM shall also allow documenting the differences across the districts/states and the potential reasons for comparison and interpretation. The formative evaluation (FE) approach using qualitative enquiry and documentation shall inform the explanation and outcome attribution (14). The FE can (a) highlight actual vs. planned interventions, (b) enable implementation through the identification of modifiable barriers, (c) facilitate any needed refinements in the original implementation intervention, (d) enhance the interpretation of project results, and (e) identify critical details and guidance necessary for replication of results in other settings.

#### 2.9.2 Evaluation approach

We propose the following evaluation approaches for the proposed IR.

##### 2.9.2.1 Baseline and endline assessments

Baseline and Endline assessments will be conducted to assess the impact and implementation outcomes. The key impact indicators related to the identification and management of HRPs. This shall be conducted at the community level through household surveys. In addition, stakeholders' qualitative assessments (Focus Group Discussions, In-Depth Interviews) will be conducted to understand the fidelity and adaptation of the implementation model.

In addition, a structured questionnaire assessing knowledge, attitudes, and practices (KAP) related to HRP identification and care will be administered to all ASHAs and ANMs. A pre-training survey will be conducted before the training, and a post-training survey will be conducted after the training to measure changes in KAP. The questionnaire will be developed based on formative research findings and will be refined through expert review. Endline assessment for pregnant women will use a semi-structured questionnaire to collect demographic information, antenatal, intrapartum, and postnatal care details, along with their feedback on the services received at the health facility.

##### 2.9.2.2 Concurrent assessment

The pregnancy coverage and quality of care indicators will be assessed concurrently and cumulatively every 6 months to monitor progress and inform the refinement of implementation strategies. This data may be collected from various sources, including primary data collected by the research team from end-users, health system/HMIS (Health Management Information System) data/PCTS (Pregnancy, Child Tracking & Health Services Management System), and other sources. Data quality assurance measures will include triangulation with other RCH registers, independent verification by field supervisors, and sensitivity analyses to assess the impact of potential data limitations on results.

##### 2.9.2.3 Process-monitoring

The research team will document implementation processes by measuring process indicators (Table 2).

**Table 2:** Process and outcome indicators for the Continuum of maternal care.

Process	Indicator
Identification of pregnant women	• Proportion of pregnant women identified by ASHA in the first trimester.
Check-ups and Care services during antenatal care visits	• Proportion of identified pregnant women who received first antenatal care in the first trimester. • Proportion of pregnant women who received all the planned check-ups (hemoglobin, BP, blood sugar, gestational age, weight gain, and other diagnoses) during antenatal care visits. • Proportion of pregnant women who received TT injection and 180 IFA tablets.
Identification and management of high-risk pregnancy	• Proportion of pregnant women identified with high pregnancy conditions. • Proportion of identified high-risk pregnancies monitored by the ASHA. • Proportion of pregnant women identified with high pregnancy conditions received appropriate care.
Referral	• Proportion of high-risk pregnancies identified referred for further management
Intrapartum care	• Proportion of deliveries conducted using a safe birth checklist. • Proportion of HRPs received appropriate care (PIH, severe anemia, APH, PPH, pre-eclampsia, preterm labour). • Proportion of pregnant women with impending obstruction/asphyxia managed by c-section timely.
Postpartum care	• Proportion of women who received scheduled postpartum contact. • Proportion of HRPs with satisfactory maternal and infant outcomes after 42 days after birth.
Outcome indicator	• Proportion of identified pregnant women with four or more antenatal care visits per schedule.

##### 2.9.2.4 Ethics review

The project will be submitted to the institutional committee of ICMR NIIRNCD, Jodhpur, for its clearance.

### 2.10 Data analysis

The study will utilize both qualitative and quantitative methods to get a complete understanding of maternal health.

#### 2.10.1 Qualitative data

Thematic analysis will be used for the qualitative data to identify the barriers and facilitators in implementing existing national programs, namely the PMSMA and PMMVY, LaQshya and SUMAN and the continuum of care for high-risk pregnant women. Identify challenges in implementing high-risk pregnant women identification and continuum care, such as resource constraints, training gaps, and logistical difficulties. Highlight supportive factors that enhance high-risk pregnant women identification and continuum care, including effective protocols, skilled staff, and positive community feedback. Feedback analysis will also be used to improve implementation strategies, effectiveness of the IR and other aspects.

#### 2.10.2 Quantitative data

The quantitative data analysis approach involves descriptive and analytical methods, including tests for significance in differences between the baseline and end line. The evaluation of implementation strategies is centred around critical maternal health indicators and outcomes, covering factors such as the frequency of antenatal check-ups, modes of delivery, rates of cesarean sections, occurrences of miscarriages and stillbirths, birth weight distributions, postnatal check-up frequencies, vaccination coverage, ASHA visits, gestational weight gain patterns, hemoglobin levels, incidents of neonatal mortality, and postpartum health. Association tools like the chi-square test will scrutinize associations and identify factors affecting HRPs. Machine learning models, such as Logistic Regression and Random Forest, will be used to predict key factors influencing the identification of high-risk pregnancy (HRP). These analyze socio-demographic characteristics (age, parity), clinical variables (hypertension, anemia), ANC/PNC visit patterns, delivery outcomes and other variables.

This dual-method approach ensures a robust analysis, providing a quantitative overview and deeper insights into the multifaceted factors contributing to HRPs. Data analysis will be conducted using statistical software tools such as R and SPSS for quantitative analysis and NVivo or Atlas.ti for qualitative analysis.

### 2.11 Timeline of the study

Table 3.

**Table 3:** Timeline of the study.

Phase	Duration	Description
Formative phase	6 months	This phase consists of a mixed-method data collection (qualitative and quantitative) and a literature review on existing national programs, policies, and interventions related to HRPs.
Development and co-implementation of strategies in iterative cycles	9 months	This phase concentrates on the design, development, and pilot implementation of the strategy, gradually rolling out the strategies in a series of iterative cycles, allowing for continuous improvement based on practical feedback.
Full-scale implementation and exit phase	18 months	This phase includes the widespread adoption of the HRP care model across the entire study area, followed by a review of the implementation's success and planning for the project's conclusion.
Data analysis and report writing	3 months	This phase includes data analysis and report writing.

## 3 Expected outcome

The project is expected to contribute to improved coverage and quality of maternal healthcare for pregnant women in the selected district area by implementing a comprehensive set of strategies. These strategies aim to include early pregnancy registration and ensuring four high-quality ANC visits, encompassing essential components such as TT vaccinations, abdominal examinations, laboratory investigations, ultrasound scans, gestational weight gain monitoring, hemoglobin level assessments, and nutrition counseling.

Additionally, the project aim to focus on improving the identification and management of HRP, promoting institutional deliveries, and ensuring essential delivery care for mothers and newborns. By enhancing the quality and accessibility of antenatal, intrapartum, and postnatal care, the initiative will seek to improve maternal and child health outcomes. The LaQshya program may be leveraged to elevate intrapartum quality indicators during institutional deliveries.

Furthermore, the project aims to develop effective and sustainable strategies to implement and scale up evidence-based, high-fidelity, context-specific interventions to improve pregnancy and postpartum care. An integral part of the project will involve enhancing the understanding of the facilitators and barriers to implementing and sustaining these interventions. The findings and recommendations generated from this project are expected to bolster the healthcare system's capacity to provide high-quality care for high-risk pregnant women, ultimately contributing to better health outcomes in the community.

## 4 Discussion

The findings of this study emphasize the need to improve the underreporting of HRPs, particularly in developing nations like India. Nearly half of all women experience high-risk pregnancies; however, initiatives such as the PMSMA underreported these cases, recognizing only 14% as high-risk in India (15). This initiative has the potential to bridge the gap in both identification and continuum care, significantly obstructing efforts to enhance health outcomes for high-risk pregnancies and newborns, especially in rural regions with limited access to healthcare services.

Approximately one-third (33.7%) of the respondents effectively utilize maternal health services among women of childbearing age (16). Policymakers should prioritize interventions and ensure the uninterrupted delivery of maternal and child health (MCH) services, including prenatal care, safe childbirth, postnatal care, and child safety, even during emergencies (17). In-depth research is necessary to understand how community initiatives aimed at enhancing support for maternal care impact the health outcomes of individual pregnant women (18). Our research aims to highlight several crucial areas for intervention and enhancement in managing HRPs by utilising existing maternal health services.

First, establishing and optimizing comprehensive antenatal, intrapartum, and postnatal care services through PHCs requires a robust institutional framework supported by effective governance and systematic coordination (19).

Secondly, the active involvement of community organizations and engagement of local stakeholders are essential for successfully implementing and utilising these services. Collaborating with educational institutions, PHCs, health departments, and partnerships involving NGOs and community-based organizations can significantly increase the reach and effectiveness of interventions targeting HRPs.

Thirdly, the study is expected to provide valuable insights into best practices for delivering maternal health services in an effective and scalable manner. The iterative process of developing and refining these models will involve ongoing feedback and adjustment, ensuring that the interventions remain relevant and impactful throughout their deployment. Significant progress can be made toward reducing maternal morbidity and mortality by addressing these challenges, thereby enhancing overall health outcomes (20).

Anticipated limitations must also be acknowledged. Some challenges may affect this study's implementation and interpretation. Implementation bias may occur due to inconsistent intervention delivery across three PHCs from varying staff training and motivation. ANMs and ASHAs may resist new workflows due to heavy workloads. Findings may not generalize beyond Nagaur due to Rajasthan's unique socio-cultural and infrastructural factors.

## 5 Conclusion

In summary, enhancing the healthcare delivery system for HRPs through Primary Health Centres offers a valuable strategy for identifying and managing HRPs in India. This implementation research will deliver a practical package in the three selected through refresher training of all ASHAs and ANMs, strengthened facility- and community-level awareness for pregnant women and their families, and regular monthly PMSMA camps with visiting obstetrician/gynaecologist support. Achieving this goal needs a collaborative effort to strengthen institutional frameworks, engage communities, and create strategies tailored to specific contexts. By addressing the existing gaps in the identification and continuity of care for HRPs, particularly those exposed by inconsistencies in reporting high-risk cases, this initiative seeks to develop and implement context-sensitive strategies within established national programs. The study will result in markedly improved identification of high-risk pregnancies, better follow-up and specialist consultation for affected women, higher completion of recommended antenatal visits, increased institutional deliveries among HRPs, and strengthened continuum of care from pregnancy through the postnatal period.

Additionally, the iterative process of collaboratively developing and refining these health service delivery models ensures that the interventions remain pertinent and adaptable to the distinct needs of various communities. Because the entire package uses only existing government staff and existing PMSMA platforms, the refined model will be fully sustainable and immediately adoptable by the routine health system.
